# Differences of Soil Fertility in Farmland Occupation and Supplement Areas in the Taihu Lake Watershed during 1985–2010

**DOI:** 10.3390/ijerph110605598

**Published:** 2014-05-26

**Authors:** Weizhong Su, Gaobin Ye

**Affiliations:** 1 State Key Laboratory of Lake Science and Environment, Nanjing Institute of Geography and Limnology, Chinese Academy of Sciences, 73 East Beijing Road, Nanjing 210008, China; E-Mail: xiegb0000@163.com; 2 University of Chinese Academy of Sciences, No. 19A Yuquan Road, Beijing 100049, China

**Keywords:** areas of farmland occupation and supplement, soil fertility, differences, farmland policy, Taihu Lake watershed

## Abstract

Since the 1980s a series of farmland policies have been implemented in China to stabilize the balance of farmland quantity and quality against accelerating urbanization and industrialization processes. This paper aims to reveal differences of soil fertility in the farmland occupation area (FOA) and farmland supplement area (FSA). In 1985–2000 the decline of the FOA area was 181,000 ha, but the FSA rarely increased. In 2000–2010 the decline of the FOA area was 824,800 ha, but the FSA increased dramatically. The accelerating loss process is closely related to urbanization and industrialization of the locations. Most occupied farmland was still located in the areas with higher soil fertility. The FOA in 1985–2000 had higher soil fertility than the FSA, but the FSA in 2000–2010 significantly raised its soil fertility to close to the FOAs’ level. The rate of excellent-good levels of the FOA in 2000–2010 decreased from 46.13% to 37.61%; The development model shifts and farmland policies implementation are the chief driving factors behind AFOS changes. The TDBF policy and the main function zoning project should continue to play an effective role in balancing the farmland system.

## 1. Introduction

The World’s urban-dwelling population has increased rapidly since the end of the 19th century and overall percentage has risen from 13% in 1900 to 49% in 2005, a figure expected to reach 60% by 2030. The negative environmental impacts associated with urbanization, such as loss of cultivated land and biodiversity are linked to the patterns of land use, specifically unwieldy and low-density urban sprawl, which has increased at alarming rates in many countries worldwide. The statistical data from the Food and Agriculture Organization (FAO) of the United Nations showed that the World’s cultivated land area was 1.401 billion hectares in 1990, and it dropped to 1.381 billion hectares in 2008; with the continued population growth, the World’s cultivated land *per capita* was 0.265 ha in 1990, and it dropped to 0.205 ha in 2008 [1]. Having enough cultivated land to feed the World population in 2050 is a major challenge, and it is a meaningful task to explore the cultivated land dynamics for the major countries of the World. Among the different situations, population growth and economic development have been two of the key driving forces for the cultivated land changes. However, due to different land use potentials and different degrees of political stability, the factors influencing cultivated land changes are different among the referenced counties [2,3,4]. For Bangladesh, Japan, Russia and the United States, urbanization and industrialization are the main reasons behind the reduction of cultivated land. However, because different countries have different reserve land resources, the cultivated land growth rate among these countries is significantly different [5].

The Taihu Lake watershed, always known as the traditional high-yield grain-producing region of China, not only had self-sufficient supplies but also exported grain to other regions. The land consumption and sprawl in the Taihu Lake watershed has outpaced population growth under different urbanization development models, such as the Sunan model (Sunan refers to a typical region mostly including Suzhou, Wuxi and Changzhou) in the 1980s and New Sunan model in the 1990s [6]. The Sunan model obviously accounted for the construction land sprawl and industrial economic development of enormous small towns, but in the 1990s, especially after 2000, facing the problems of severe eco-environmental and land resource shortages, a new Sunan model came forward and presented strongly developing big cities and Development Area economies. Big-cities’ fringe areas, such as those of Shanghai, Changzhou, Wuxi and Suzhou, had to start transforming cultivated land into urban land. More recently, the Taihu Lake watershed has been the forefront of urbanization and industrialization processes in China, and it is predicted to be the biggest megalopolis in the whole Yangtze River delta and China, but had a large amount of farmland was replaced by artificial ecosystems. Although the population in the region accounted for 2% of the population of the whole country in 2010, its gross domestic product (GDP) of up to 10.17 million RMB has been 7% of the national GDP, and the GDP *per capita* also is more than three times than that of the whole country. However, landscape change associated with urbanization, particularly urban sprawl, has brought about a farmland decline in the whole area, and the serious farmland loss has become an obstacle to the sustainable development of these areas [6,7]. In 2010 the farmland area *per capita* was only 0.04 ha, not only lower than the security value of 0.053 ha recommended by the FAO but also only a half of the 0.092 ha of farmland *per capita* in the whole country [8].

To solve these serious problems, the Total Dynamic Balance of Farmland (TDBF) in policy of the *Land Management Law* issued by the Chinese government in 1999, also known as the balance of the areas of farmland occupation and supplement (AFOS) in the total area within an administrative region in a certain period, began to be implemented in the whole country in order to control the farmland loss, and required that the total amount of farmland supplement area (FSA) to be increased by land reclamation, consolidation or development, must be not less than that of farmland occupation area (FOA) decreased due to urban sprawl and industry development. The policy of TDBF significantly increased the FSA, reduced the FOA, and relieved the pressure of farmland loss.

However, both facts and research have indicated that while the TDBF policy narrowed the gap in quantity between the FOA and FSA, the FSA quality was still far from the FOA quality [9,10]. Firstly, from the viewpoint of location conditions, the FOA was mainly located on the plains around cities and along roads, but the FSA in 1985–2000 largely happened in mountainous and rural areas. The incremental farmland had poor crop growth conditions and low degree of topsoil maturation and significantly decreased grain productivity. Secondly, the FSA checks by official agencies also only considered some visual indices, such as topsoil thickness, irrigation conditions and the leveling of soil *etc.*, which easily result in differences in the quality of the FSA and FOA [11]. Wang and Qu [12] used intuitive indices like the crop yield and production cost to conclude that the overall quality of farmland tended to decline during 1999–2001 with the TDBF policy implementation, but we think that their method only measured the farmland output capacity, which was yet influenced by other factors too, such as plant species, the chemical fertilizer dosage, irrigation conditions, *etc*. The soil fertility is only one of the most important components of farmland quality. Chen [13] and Li *et al.* [14] used the second Census Data of China National Soil (CDCNS_II_) and found that the non-agricultural land in 1984–2003 easily occupied the farmland with higher soil quality in urbanization areas, but the incremental farmland happen preferentially rural areas with low soil quality. Thus the quality of the FSA is significantly lower than that of the FOA, and the TDBCL policy only reflected a balanced quantity of farmland occupation and supplement. Then in 2006 the method of farmland gradation conversion coefficient was proposed and implemented in the whole country in order to solve the quality unbalance between the FOA and FSA. According to the principle of maintaining stable farmland production capacity, the policy made a gradation equivalent conversion through projects of land development, reclamation and consolidation. The outline of the latest Land-use Planning proposed that the FSA should mainly come from the land consolidation and reclamation of construction land and significantly promoted farmland quality [15].

Thus, since the 1980s the farmland changes in the Taihu Lake watershed have passed through four key issues and the corresponding phases of farmland policy: farmland loss pressure-the TDBF policy control-the farmland quality unbalance-the land gradation conversion method. Existing studies have mostly focused on the soil fertility of the FOA in some areas of the watershed before 2000, but this paper regarded the whole watershed as the study unit to measure differences in soil fertility between the FOA and FSA, and also fully considers the effect of new regional development and farmland protection policies on farmland changes.

## 2. Data and Method

### 2.1. The AFOS Changes

Landsat-MSS acquired on 10 April 1985 and Landsat-TM of 17 April 17 2000 and 22 April 2010, were used to extract land use/cover information based on the composition of bands 5, 4 and 3. Three images were acquired in sunny weather, so there was no need to perform atmosphere corrections. Firstly, the images were corrected using 1:100,000 topographic maps with 150 control points for each image. All images were resampled to a common nominal spatial grid of 25 m resolution using the nearest neighbor technique, which would be required for the change detection analysis. The root mean square errors of resampling and reprojection of the images were less than 0.5 pixels, equivalent to approximately 7–15 m. In this study, the software package, eCognition Definiens Developer 7.0 was used to implement object-oriented classification, Overall accuracy (OA) and kappa coefficient (Kappa) are used to evaluate the classification results. The results have indicated that the OA, Kappa of the classifications were 86.22%, 0.844, and 88.77%, 0.93, and 89.11%, 0.89 for images in 1985, 2000 and 2010, respectively. The paper collapsed them into ten types of land use: farmland, woodland, garden land, grassland, water (rivers and lakes), wetland (ponds, swamps and beaches), urban land, rural land, industrial-mining land, unused land, among which urban, rural and industrial-mining land are often regarded as the construction land.

**Figure 1:**
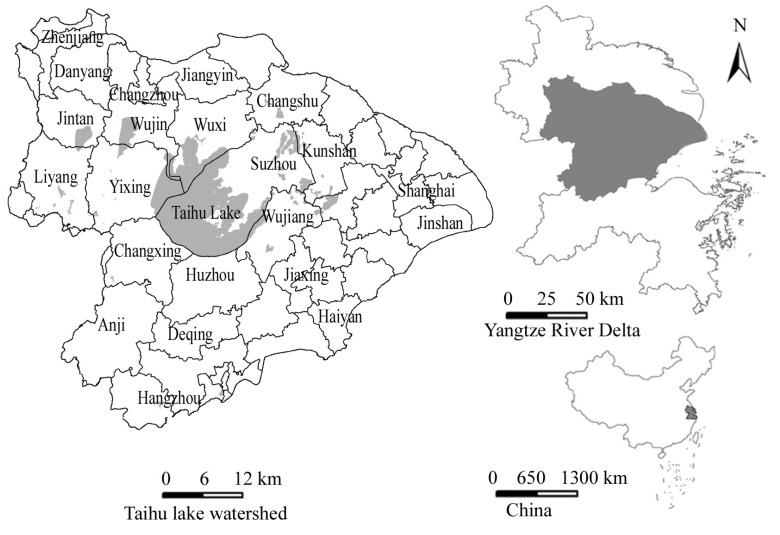
The location of the study area.

Under the overlay analysis in the ArcGIS software, the farmland layers of 1985 and 2000 were intersected into the FOA and FSA layers during 1985–2000, and the farmland layers in 2000 and 2010 into the two FOA and FSA layers during 2000–2010. Then by intersecting AFOS layers, other land types and soil fertility data, we could get land transformation matrix and also the soil fertility features in the AFOS (Figure 1).

### 2.2. The Soil Fertility of AFOS

The Census Data of China National Soil throughout the county in China included the CDCNS_I_ (1958–1960) and CDCNS_II_ (1979–1985). We used 1:500,000 soil nutrient maps derived from the Data Exchange and Sharing Platform of Lake and Watershed of Chinese Sciences Academy. Soil fertility provides nutritional and coordinate environmental conditions for crops growth and is measured by using CDCNS_II_ data of the study area and several common parameters, such as nutrients, texture, pH, *etc*. [16]. First, the soil texture, known as relatively stable soil attributes, refers to the soil particle size and commonly includes sand, loam and clay and the other types. It affects not only the soil oxygen content, redox properties and ventilation condition but also the nutrients conversion rates of the soil and the physiological activities of plant root growth. Due to the different growth habits of crops, clayey soil is more suitable for rice but sandy soil is more suitable for wheat. Hence the structural value of soil texture for each soil patch unit is calculated by multiplying the proportion of soil texture types in the CDCNS_II_ data and the corresponding weight suitable for the specific crop. The weight of the clay, loam and sand texture for the rice and wheat, respectively, are 0.42, 0.37, 0.21 and 0.37, 0.42, 0.21 [11]. Then we divide all soil patch units into five levels based on their structural value by the method of the natural breaking point in the ArcGIS software. Second, the pH value in the range 6.5–7.5 is most suitable for crops growth and also is divided into five levels suitable for the dominant crops, which are the rice in most of the study area and prefer an acidic soil to an alkaline soil [6] (Table 1). 

**Table 1:** The standard of soil nutrients and pH classification.

		Excellent	Good	Fair	Common	Poor	Weight
**Nutrients**	OM%	>4	3–4	2–3	1–2	<1	0.422
	TN%	>0.2	0.15–0.2	0.1–0.15	0.07–0.1	<0.07	0.356
	TP%	>0.2	0.15–0.2	0.1–0.15	0.05–0.1	<0.05	0.222
**pH**		6.5–7.5	7.5–8	5–6.5	0–5	>8	-

Last, the soil nutrients, mainly including the organic matter (OM), total nitrogen (TN) and total phosphorus (TP), directly affect the transformation, release and the validity of reserves of soil nutrients in the crop growth process. We set the classification standard by referring to related studies in the province of Jiangsu, Zhejiang and Shanghai city [17,18], then calculated the weight value of each nutrient parameter by using the factor analysis method in the SPSS software, and also divided the results into five levels based on the weighted sum method (Table 1).

## 3. Results and Discussion

### 3.1. The AFOS Change Features

**Figure 2:**
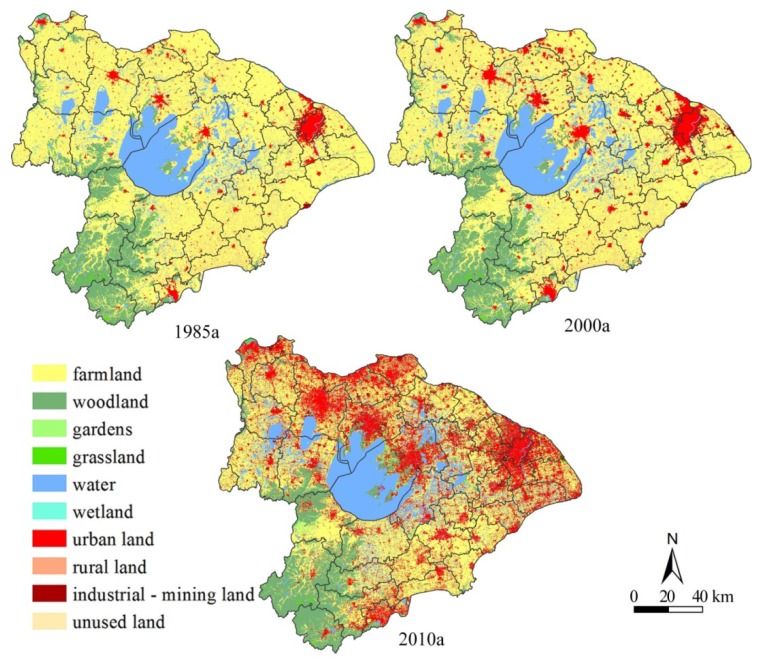
The spatial pattern of AFOS change.

Especially since 2000, the farmland in the Taihu Lake watershed, with a total area of 3,688,440 ha, has sustained an accelerating loss process, and the percentage of farmland in 1985, 2000, and 2010 declined from 63.84% to 58.95% to 43.94%. The average decline of farmland area in 1985–2000 was 12,040 ha, equivalent to 0.51% of the total farmland area in 1985. However, the average decline of farmland area in 2000–2010 rose to 55,340 ha, equivalent to 2.55% of the total farmland area in 2000 according to Table 2. In 1985–2000 the decline of the FOA area was 181,000 ha, but the FSA rarely increased and was only 2,555 ha. In 2000–2010 the decline of the FOA area was 824,800 ha, but the FSA increased dramatically and rose to 271,400 ha. The geomorphologic features and a long cultivation history in the study area are dominant factors determining the high percentage of farmland in the whole watershed. Plains cover nearly all of the watershed, including the Hangzhou-Jiaxing-Huzhou plain, the western Taihu plain and the plain along the Yangtze River (Figure 2). Additionally, although the farmland decreased rapidly after 2000, these plains have served as the traditional grain producing areas and still occupied the dominant proportion of farmland in the region [19].

**Table 2:** The AFOS change features.

Years	1985	2000	2010
Farmland area (ha)	2,354,700	2,174,100	1,620,700
Farmland percentage (%)	63.84	58.95	43.94
Construction land area (ha)	2,354,700	2,174,100	1,620,700
Construction land percentage (%)	63.84	58.95	43.94

The AFOS features and farmland transformation matrix during 1985–2000 showed that the FOA area was up to 181,000 ha, 89.1% of which had been transformed into construction lands in urban fringe areas (urban, rural, industrial-mining land), and 7.85% into wetlands, and 2.38% into woodlands, and the remaining less than 0.7% into other uses. The FSA area came mainly from the development of mountainous woodlands and unused land in rural areas and was only 2,555 ha, 43.3% of which came from woodlands, and 22.28% from rural and industrial-mining lands, and nearly 30% from wetlands, garden, grasslands and unused lands (Table 3). 

**Table 3:** The AFOS transformation matrix.

Land Use Patterns	FOA 1985–2000		FSA 1985–2000		FOA 2000–2010		FSA 2000–2010	
	Area (ha)	Area %	Area (ha)	Area %	Area (ha)	Area %	Area (ha)	Area %
Woodland	4,306.92	2.38	1,089.05	43.30	102,114.56	12.38	56,667.45	20.88
Garden	392.95	0.22	264.46	10.52	43,032.76	5.22	9,147.14	3.37
Grassland	17.66	0.01	86.42	3.44	2,396.73	0.29	2,281.90	0.84
Water	794.86	0.44	35.78	1.42	3,623.03	0.44	11,815.39	4.35
Wetland	14,199.10	7.85	411.31	16.35	31,727.19	3.85	38,708.79	14.26
Urban land	82,034.76	45.33	33.64	1.34	324,423.64	39.33	20,228.60	7.45
Rural land	69,844.87	38.60	271.47	10.79	225,316.68	27.32	127,610.68	47.02
Industrial-mining land	9,356.61	5.17	289.04	11.49	92,137.98	11.17	4,653.26	1.71
Unused land	13.26	0.01	33.82	1.34	60.46	0.01	256.79	0.09
Summary	180,961	100	2515	100	824,833	100	271,370	100

The above features during 2000–2010 showed that the FOA entered an obviously accelerating phase and was transformed into not only construction lands but also other land types. The FOA area increased to 824,800 ha which was 4.56 times larger than that in 1985–2000, 77.8% of which had been transformed into construction lands, and 12.38% into woodlands, and 9.07% into wetlands and gardens. Then, the FSA area increased dramatically throughout the watershed, up to 271,400 ha which was 100 times larger than that in 1985–2000, and this was mainly from the consolidation and reclamation of construction lands (accounted for 54.5%) and the development of grasslands, wetlands and unused land (total rate up to 35%).

### 3.2. The AFOS Soil Fertility Features

**Figure 3:**
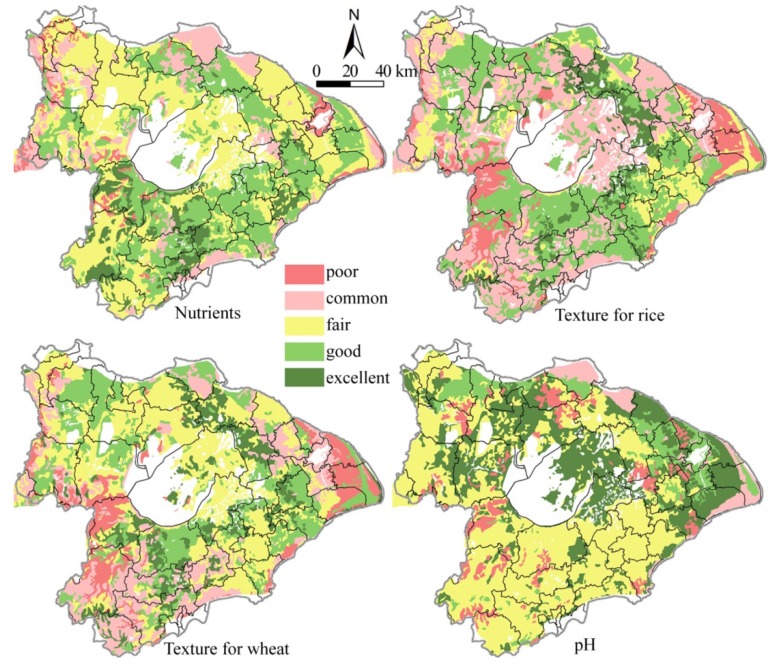
Soil fertility classification patterns.

Figure 3 and Table 4 show that most of the soils in the study area were suitable for crop growth, as the percentage of excellent-good levels of both soil nutrients and texture were about 40% and the percentage of the soils with pH values in the 6.5–7.5 range was also up to 31.88%. Excellent-good levels of soil nutrients are largely located at the belt area along Taihu Lake and the Shanghai-Nanjing traffic corridor, and the fair level of nutrients corresponds to a larger area adjacent to the locations with excellent-good levels, and scattered areas with poor levels of nutrients are along the Yangtze River and mountains in the watershed. The areas of Changzhou-Wuxi-Suzhou-Jiaxing-Huzhou along Taihu Lake and along the Shanghai-Nanjing traffic corridor are both suitable for the growth of wheat and rice, but the southern Liyang and the area along the Jiaxing-Haiyan line is more suitable for wheat growth. Summarily, most soils are suitable for crop growth and are located in the areas along Yangtze River, Shanghai-Nanjing traffic corridor and Shanghai-Hangzhou traffic corridor, in which urban sprawl and the development of Economic Development Area occupied a large amount of farmland rapidly in Taihu Lake watershed.

**Table 4:** Soil fertility levels structure.

Levels Name	Excellent	Good	Fair	Common	Poor
Nutrients levels standard	>4	3–4	2–3	1–2	<1
Area (ha)	274,900	1,036,200	1,404,700	431,500	142,200
Area %	8.36 >34.95	31.50 33.53–34.95	42.70 31.82–33.53	13.12 28.69–31.82	4.32 <28.69
Texture for rice levels standard					
Area (ha)	391,400	755,900	1,159,000	656,100	327,100
Area %	11.90	22.98	35.23	19.94	9.94
Texture for wheat levels standard	>34.95	33.53–34.95	31.82–33.53	28.69–31.82	<28.69
Area (ha)	329,600	1,048,000	395,900	1,184,500	331,600
Area %	10.02	31.86	12.04	36.01	10.08
pH levels standard	6.5–7.5	7.5–8 ^1^	5–6.5 ^2^	>8	<5
Area (ha)	1,048,600	259,700	1,691,900	126,500	162,900
Area %	31.88	7.89	51.43	3.85	4.95

Notes: Both ^1^ and ^2^ refer to good level, and ^1^ for the wheat, and ^2^ only for the rice.

In 1985–2000 the FOA had higher soil fertility than the FSA, and the rate of excellent-good levels of the FOA soil fertility was 7% points higher than that of the FSA soil fertility. The actual area of the FOA was 181,000 ha, and that of the FSA only 2,555 ha, among which the area of excellent-good levels of the FOA was 83,300 ha and that of the FSA only 900 ha. The area of excellent-good levels in the net loss of farmland was up to 82,300 ha, that is, the annual net loss area was up to 5,500 ha, which accounted for the 46.13% of the whole net farmland loss in the same phase. However, the rate of excellent-good levels of FOA soil fertility in 2000–2010 had a drop of 4.25% points, but the same index of the FSA soil fertility had an increase of 5% points. Therefore, due to the significant improvement in FSA soil fertility after 2000, the gap of soil fertility between FOA and FSA had declined from 7% points in 1985–2000 to 1.25% points in 2000–2010. The actual area of the FOA was 824,800 ha, and that of the FSA dramatically increased to 271,400 ha. The area of excellent-good levels in the net loss of farmland was up to 208,100 ha, that is, the annual net loss area was up to 20,810 ha and accounted for 37.61% of the whole net farmland loss.

Thus, since 2000 the net loss of farmland continued to increase and the avarage area of excellent-good levels also increased from 5,500 ha to 20,800 ha, but the gap of soil fertility in the FOA and FSA was narrowing due to the significant improvement of FSA soil fertility, and the rate of excellent-good levels of the net loss of farmland also obviously decreased from 46.13% before 2000 to 37.61% after 2000 (Table 5).

**Table 5:** The percentage of AFOS soil fertility levels.

Fertility Parameter	AFOS	Excellent	Good	Fair	Common	Poor	Others
Nutrients	FOA 1985–2000	11.12	34.87	43.31	8.69	2.01	0
	FSA 1985–2000	2.11	34.58	36.89	17.89	6.54	1.99
	FOA 2000–2010	9.63	33.02	41.89	12.41	3.05	0
	FSA 2000–2010	11.98	42.17	27.72	10.32	4.81	3
pH	FOA 1985–2000	32.82	29.42	11.63	11.41	14.72	0
	FSA 1985–2000	41.72	15.32	21.85	6.31	13.8	1
	FOA 2000–2010	20.61	20.15	27.62	16.21	15.41	0
	FSA 2000–2010	20.54	17.02	35.86	11.26	12.32	3
Texture for rice	FOA 1985–2000	18.66	27.05	31.98	13.35	8.96	0
	FSA 1985–2000	14.55	23.33	30.72	13.43	15.97	2
	FOA 2000–2010	21.87	27.12	30.73	10.15	10.13	0
	FSA 2000–2010	22.87	19.13	32.56	15.36	7.18	2.9
Texture for wheat	FOA 1985–2000	15.59	32.15	20.88	20.35	11.03	0
	FSA 1985–2000	13.85	28.11	20.81	19.24	13.99	4
	FOA 2000–2010	20.96	31.12	21.92	16.31	9.69	0
	FSA 2000–2010	18.76	26.04	12.97	29.08	10.88	2.27

## 4. Discussion

### 4.1. Driving Factors of the AFOS Change Features

The accelerating farmland loss process is closely related to the placements of the urbanization and industrialization development. Most plain areas in the Taihu Lake watershed almost cover all the urban and industrial agglomeration belts along the Yangtze River, Shanghai-Nanjing traffic corridor and Shanghai-Hangzhou traffic corridor, in which urban sprawl and the development of the Economic Development Area (New Sunan model) intensely occupied a large amount of farmland [20]. Figure 2 and Table 2 showed that the construction land mostly happened at areas around the cities and Economic Development Areas, and the percentage of construction land increased from 9.73% in 1985, to 14.22% in 2000, and 25.88% in 2010.

Nearly 90% of the FOA in 1985–2000 had been replaced by urban, rural and industrial-mining land around cities and towns under the drive of Sunan model and was grouped into three categories. One refers to the rapid decrease of farmland around large cities, such as Pudong, Songjiang districts of Shanghai, southeast Suzhou, along the Shanghai-Nanjing traffic corridor of Wuxi, Wujin and Xinbei of Changzhou. These cities, as the main centers of the modern economy, play a critical role for economic development and also have to take up much farmland resources. The second category refers to some scattered FOA patches around the counties and towns, such as Zhenjiang, Kunshan, Changshu, Zhangjiagang, Jiaxing, Yixing and Liyang under the Sunan model. The third category refers to the start of farmland occupation in rising Economic Development zones, which, also as a national development strategy, arose in the early 1990s. The remaining FOA had been occupied by wetlands and woodlands. Aquaculture is one of main industrial activities in the water villages of the Taihu Lake watershed. Also, the conversion of farmlands into woodlands in the suburbs of larger cities and water reservoirs is closely related to the national implementation of the policy of Returning Farmland to Forests proposed in 1998 [21]. However, the FSA patches in 1985–2000 were concentrated in remote rural areas around Shanghai, Zhenjiang, Changzhou, Wuxi, Suzhou, in which peasants had spontaneous demands of developing farmland from unused land due to the rapid farmland loss in these developed areas. Thus, the new farmland was largely unused wetlands and woodlands land along rivers and mountains, but the woodlands in the Zhejiang Mountain areas have still been less occupied by farmland due to the low farmland demand and high development costs.

In 2000–2010, the accelerating urbanization and industrialization processes, economic structural adjustment and farmland policy implementation started to be dominant factors affecting AFOS transformation changes. Firstly, the regional development model had shifted from the Sunan model to the new Sunan model, and big cities and Economic Development Areas increased dramatically to occupy a large amount of farmland but the speed of the farmland loss in rural areas slowed down relatively. Secondly, the policy of returning farmland to forests implemented in 1999–2004 resulted in the conversion of some farmlands into woodlands around the cities and along the mountains, lake and rivers. Thirdly, aquaculture ponds and suburban nursery orchards (gardens) as the emerging leisure industry were raising along the Shanghai-Nanjing traffic corridor and replaced some farmland [22]. The modern tourism at mountains, lakes and islands was also increasing with the leisure habits of urban residents and this also converted farmlands to construction lands [23]. Lastly, there was a rapid increase of the FSA throughout the watershed due to the full implementation of the Dynamic Balance of Farmland policy throughout the country. Summarily, the differences of the development models and farmland policies in the Taihu Lake watershed around 2000 are the principle driving forces of the AFOS feature changes, but the amount of FSA is still far below that of the FOA and the dynamic balance of farmland has still not been met.

### 4.2. Driving Factors of the AFOS Soil Fertility Differences

Better fertile soil spatially covers most city areas, and then construction activities and urban expansions under the effect of urban agglomeration economy make it difficult to avoid occupying the high-quality farmland. As everyone knows, human settlements often originate in river alluvial plains with fertile soil and flat terrain suitable for cultivation. Again, a long cultivation history with excellent conditions in sunlight and heat make farmland more fertile [24,25]. 

In 1985–2000, the chief reason of most FOA with higher fertile soil is because the farmland has been largely occupied by construction activities around urbanization areas with fertile soil resources in the plain. But the FSA mainly came from the development of unused land and woodlands in rural and mountain areas with poor location conditions and low maturation of soils [11,26]. In 2000–2010, the FOA was largely converted into construction land, but the conversation sources of FSA began to shift from the previous development of unused land and woodlands into the consolidation and reclamation of construction land with excellent location conditions and high maturation of soils [27]. In 2000–2010, as 54.5% of the FSA, nearly 147,800 ha, was from the construction consolidation and reclamation (Table 3), the method of agricultural land gradation conversion coefficient in 2006 played a direct role in the soil fertility improvement of the FSA.

### 4.3. Application of Policies for Future Development

Firstly, the expansion of construction activities around cities and Economic Development areas are the chief factors driving farmland loss with higher fertility soil, and the policies of the TDBF and agricultural land gradation conversion coefficient have played an effective role in balancing the quality and quantity between the FOA and FSA. In the context of fully implementing a national urbanization strategy, construction activities and expansions would still dominate the regional development direction in future. Thus, to adhere to the principle of balancing the farmland it is essential to ensure the productivity of farmland in the Taihu Lake watershed.

Secondly, the main function zoning, a strategic, basic, compulsory planning, is a giant project proposed by the government and aims to guide urban and land-use planning in China. According to resources and environment carrying capacity of natural ecosystems, the existing development density and development potential of human-made ecosystems, the main function zoning (MFZ) plans future population distribution, economy, land use and urbanization patterns as a whole and divides land spaces into four categories areas, such as optimized development, focused development, restricted development and prohibited development areas. In the major cities, especially in the Shanghai-Suzhou-Wuxi-Changzhou-Zhenjiang areas, socio-economic and urban growth has been significant during the last twenty years and is expected to develop more rapidly in future. Therefore, the scattered and key object patches should be prohibited development areas with the response of the existing city zoning and land use planning requirements. At the same time, an urban extended boundary strictly is identified. On the other hand, the Green way system should be built by use of the rivers and the road green belts. In town areas, towns developed rapidly as the interference patches, and are still concentrated in the townships. Under the new Sunan model of regional development recently, town’s socio-economic and land growth is slowed down. In the future in these areas, a “points-axis” development model, that is, the areas around urban and along the main direction of the external transport, implies that town’s development should reduce interference with the natural ecosystem. In the area along the Yangtze River and Taihu Lake, under the guidance of the policy of riverside and shore region development of the past economic development and land growth are very rapid. In recent years, water pollution has seriously harmed the regional drinking water security. Therefore, on the one hand, the protection width of the buffer zone of two major ecological functions as prohibited development area has increased. Additionally, industrial land should be arranged in clustered spatial form. In the southern and the western mountain region as important ecological function sources, the main interference sources are from pottery industry and tourism development activities. In addition to forming a focused spatial pattern, construction land needs have a reconstruction, technological and industrial transformation function. 

Lastly, the paper underlined the comparison of FOA and FSA and also fully considered the effect of regional development and farmland protection policies on AFOS change and fertility differences. Wang and Qu [12] adopted the yield index of crops to explain soil fertility and only measured the output capacity of farmland, but the method is very incomplete because the crop output capacity is also influenced by many other factors. Chen [13] (2007) and Li *et al.* [14] used the CDCNS_II_ data and found that during 1984–2003 the non-agricultural lands in the urbanization areas were more vulnerable to occupy the farmland with higher soil fertility, while the paper used the same data to divide soil fertility into five levels and found that the percentage of the excellent-good levels of the soil fertility during 1985–2000 is 46%–62%. The different results indicated that most FOA had higher fertility soil, but should be noticed that their studies only focused on the part of the FOA in different periods. 

## 5. Conclusions

The accelerating process of farmland loss is closely related to the placement of urbanization and industrialization zones, which cover almost all the urban and industrial agglomeration belts along the Yangtze River, Shanghai-Nanjing traffic corridor and Shanghai-Hangzhou traffic corridor and intensely occupied a large amount of farmland. The farmland percentage in the whole watershed continued to decrease from 63.84% in 1985 to 58.95% in 2000 to 43.94% in 2010. During 1985–2000 89.1% of FOA areas were largely replaced by construction lands, but the FSA areas came mainly from the development of unused land in rural areas and only were 2,555 ha. During 2000–2010 the FOA areas were largely converted into not only construction lands, but also woodlands and wetlands, while the FSA were mainly from the consolidation and reclamation of construction land and increased dramatically to 271,400 ha. 

The loss of farmland is still focused on the better fertile farmland. During 1985–2000 the FOA was with better fertile soil than the FSA, but during 2000–2010 the soil fertility of the FSA was significantly raised and obviously close to the soil fertility level of the FOA. Thus after 2000 the rate of excellent-good levels of soil nutrients of the FOA decreased from 46.13% to 37.61%, but the mean annual farmland loss with excellent-good fertile levels was still from 5,500 ha up to 20,800 ha. The expansion of construction activities around cities and Economic Development areas are the basic reasons behind the loss farmlands with better fertility soil, but the TDBF policy and the agricultural land gradation conversion coefficient method have played an effective role in balancing the FOA and FSA quality and quantity.

The development models shift and farmland policies implementation in the Taihu Lake watershed are the chief factors of driving AFOS change. The TDBF policy and the agricultural land gradation conversion coefficient method have played an effective role in balancing the FOA and FSA quality and quantity and continue to play a critical role in balancing the farmland system in the context of a national urbanization strategy. At the same time, the main function zoning projects proposed by the government should determine future population distribution, economy, land use and urbanization patterns as a whole by dividing land spaces into four categories, which may vary in different regions including the major cities, town areas, the area along Yangtze River and Taihu Lake and the southern and the western mountain regions.

## References

[1] The Statistical Database of the Food and Agriculture Organization (FAO). http://faostat.fao.org/.

[2] Wirsenius S., Azar C., Berndes G. (2010). How much land is needed for global food production under scenarios of dietary changes and livestock productivity increases in 2030?. Agric. Syst..

[3] Wolf J., Bindraban P.S., Luijten J.C., Vleeshouwers L.M. (2003). Exploratory study on the land area required for global food supply and the potential global production of bioenergy. Agric. Syst..

[4] De Ridder N. (2005). Land quality, agricultural productivity, and food security: Biophysical processes and economic choices at local, regional, and global levels. Agr. Sys..

[5] Zhao W.W. (2012). Arable land change dynamics and their driving forces for the major countries of the world. Acta Ecologica Sinica.

[6] Su W.Z., Gu C.L., Yang G.S., Chen S., Zhen F. (2010). Measuring the impact of urban sprawl on natural landscape pattern of the Western Taihu Lake watershed, China. Landsc. Urban Plan..

[7] Long H.L., Liu Y.S., Wu X.Q., Dong G.H. (2009). Spatio-temporal dynamic patterns of farmland and rural settlements in Su–Xi–Chang region: Implications for building a new countryside in coastal China. Land Use Policy.

[8] Deng X.Z., Huang J.K., Rozelle S., Uchida E. (2006). Cultivated land conversion and potential agricultural productivity in China. Land Use Policy.

[9] Huang X.J., Pu L.J., Zhou F. (2002). Possibility of realizing dynamic balance of farmland area in the Yangtze River Delta. J. Nat. Resour..

[10] Huang B., Shi X.Z., Yu D.S., Öborn I., Blombäck K., Pagella T.F., Wang H.J., Sun W.X., Sinclair F.L. (2006). Environmental assessment of small-scale vegetable farming systems in peri-urban areas of the Yangtze River Delta Region, China. Agric. Ecosyst. Environ..

[11] Zhou S.L. (2004). Study on Agricultural Land Classification in Jiangsu Province.

[12] Wang F.T., Qu M. (2004). Consideration on dynamic balance maintenance of total arable land. China Popul. Resour. Environ..

[13] Chen J. (2007). Rapid urbanization in China: A real challenge to soil protection and food security. Catena.

[14] Li G.L., Chen J., Tan M.Z. (2008). Spatio-temporal impact of non-agricultural land expansion on soil resources in Suzhou City. J. Nat. Resour..

[15] Xiao B.L., Chen Y.J., Chen J. (2009). New macroscopical trends and characteristic of balance of cultivated land occupation and compensation in China. Chin. Agric. Sci. Bull..

[16] Geissen V., Guzman G.M. (2006). Fertility of tropical soils under different land use systems—A case study of soils in Tabasco, Mexico. Appl. Soil Ecol..

[17] Jiang D., Hengsdijk H., Dai T.B., De Boer W., Jing Q., Cao W.X. (2006). Long-term effects of manure and inorganic fertilizers on yield and soil fertility for a winter wheat-maize system in Jiangsu, China. Pedosphere.

[18] Huang B., Sun W.X., Zhao Y.C., Zhu J., Yang R.Q., Zou Z., Ding F., Su J.P. (2007). Temporal and spatial variability of soil organic matter and total nitrogen in an agricultural ecosystem as affected by farming practices. Geoderma.

[19] Cao Z.H., Huang J.F., Zhang C.S., Li A.F. (2004). Soil quality evolution after land use change from paddy soil to vegetable land. Environ. Geochem. Health.

[20] Su W.Z., Yang G.S., Yao S.M., Yang Y.B. (2007). Scale-free structure of town road network in southern Jiangsu Province of China. Chin. Geogr. Sci..

[21] Fan F.L., Wang Y.P., Wang Z.S. (2008). Temporal and spatial change detecting (1998–2003) and predicting of land use and land cover in Core corridor of Pearl River Delta (China) by using TM and ETM+ images. Environ. Monit. Assess..

[22] Liu Y.S., Wang L.J., Long H.L. (2008). Spatio-temporal analysis of land-use conversion in the eastern coastal China during 1996–2005. J. Geogr. Sci..

[23] Liu J.Y., Liu M.L., Zhuang D.F., Zhang Z.X., Deng X.Z. (2003). Study on spatial pattern of land-use change in China during 1995–2000. Sci. China Ser. D.

[24] Tan M.H., Li X.B., Xie H., Lu C.H. (2005). Urban land expansion and arable land loss in China—A case study of Beijing–Tianjin–Hebei region. Land Use Policy.

[25] Tian G.J., Jiang J., Yang Z.F., Zhang Y.Q. (2011). The urban growth, size distribution and spatio-temporal dynamic pattern of the Yangtze River Delta megalopolitan region, China. Ecol. Model..

[26] Yan W.J., Yin C.Q., Zhang S. (1999). Nutrient budgets and biogeochemistry in an experimental agricultural watershed in Southeastern China. Biogeochemistry.

[27] Guo H.Y., Wang X.R., Zhu J.G. (2004). Quantification and index of non-point source pollution in Taihu Lake region with GIS. Environ. Geochem. Health.

